# Supplementary material for: Chimera states in uncoupled neurons induced by a multilayer structure
Source: Sci Rep. 2016 Dec 13;6:39033. doi: 10.1038/srep39033 (PMC5153648; doi:10.1038/srep39033)
Supplement: Supplementary Information [file srep39033-s1.pdf]

Chimera states in uncoupled neurons induced by a multilayer structure

Supplementary Information

Soumen Majhi,[1] Matjaž Perc,[2,3,*] and Dibakar Ghosh[1]

[1] *Physics and Applied Mathematics Unit, Indian Statistical Institute, Kolkata-700108, India*

[2] *Faculty of Natural Sciences and Mathematics, University of Maribor, Koroška cesta 160, SI-2000 Maribor, Slovenia*

[3] *CAMTP – Center for Applied Mathematics and Theoretical Physics, University of Maribor, Krekova 2, SI-2000 Maribor, Slovenia*

*Corresponding author: matjaz.perc@uni-mb.si

In this Supplementary Information, we briefly discuss the following:

I. Formation of chimera state in the upper layer of uncoupled neurons in presence of non-local interaction among the neurons of the lower layer (the multi-layering layer).

II. Chimera pattern in the proposed multi-layer structure in presence of inter layer heterogeneity.

I. **Formation of chimera state in the upper layer of uncoupled neurons in presence of non-local interaction among the neurons of the lower layer (the multi-layering layer)**

We consider non-local interaction in the lower layer where both types of synapses (electrical and chemical) are present as proposed in the article. With instantaneous inter layer chemical synaptic coupling, the equations governing the dynamics of upper layer are as follows:

$$\begin{aligned}
\dot{x}_{i,1} &= a{x_{i,1}}^2 - {x_{i,1}}^3 - y_{i,1} - z_{i,1} + K_{ch}(v_s - x_{i,1})\Gamma(x_{i,2}(t)),\\
\dot{y}_{i,1} &= (a+\alpha){x_{i,1}}^2 - y_{i,1},\\
\dot{z}_{i,1} &= c(bx_{i,1} - z_{i,1} + e),
\end{aligned} \tag{1}$$

and for the lower layer as the following:

$$\begin{aligned}
\dot{x}_{i,2} &= a{x_{i,2}}^2 - {x_{i,2}}^3 - y_{i,2} - z_{i,2} + K_{ch}(v_s - x_{i,2})\Gamma(x_{i,1}(t)) + K_{el}\sum_{j=i-P, j\neq i}^{i+P} E(x_{j,2}, x_{i,2}),\\
\dot{y}_{i,2} &= (a+\alpha){x_{i,2}}^2 - y_{i,2},\\
\dot{z}_{i,2} &= c(bx_{i,2} - z_{i,2} + e),
\end{aligned} \tag{2}$$

Square-wave bursting dynamics is assumed for all the nodes of the two layers with the set of parameter values: $a = 2.8, \alpha = 1.6, c = 0.001, b = 9$, and $e = 5$. The chemical synaptic coupling function $\Gamma(x)$ is the following

$$\Gamma(x) = \frac{1}{1+e^{-\lambda(x-\Theta_s)}}, \tag{3}$$

whereas $E(x_{j,2}, x_{i,2}) = x_{j,2} - x_{i,2},\ i, j = 1, 2, ..., N$ with $v_s = 2.0$, $\Theta_s = -0.25$ and $\lambda = 10$, as before. Here P is the number of coupled nearest neighbor neurons on both sides on a ring.

At first, we fix the electrical synaptic coupling strength $K_{el} = 2.0$ and vary the number of nearest neighbors P and chemical synaptic coupling strength K_{ch} to observe the behavior of uncoupled neurons. Taking $P = 10$ for which with $K_{ch} = 0.0$, the lower layer of neurons interacting through electrical coupling gets synchronized. Then switching K_{ch} on, we observe chimera state in the upper layer of uncoupled neurons. Typical snapshots for both the upper

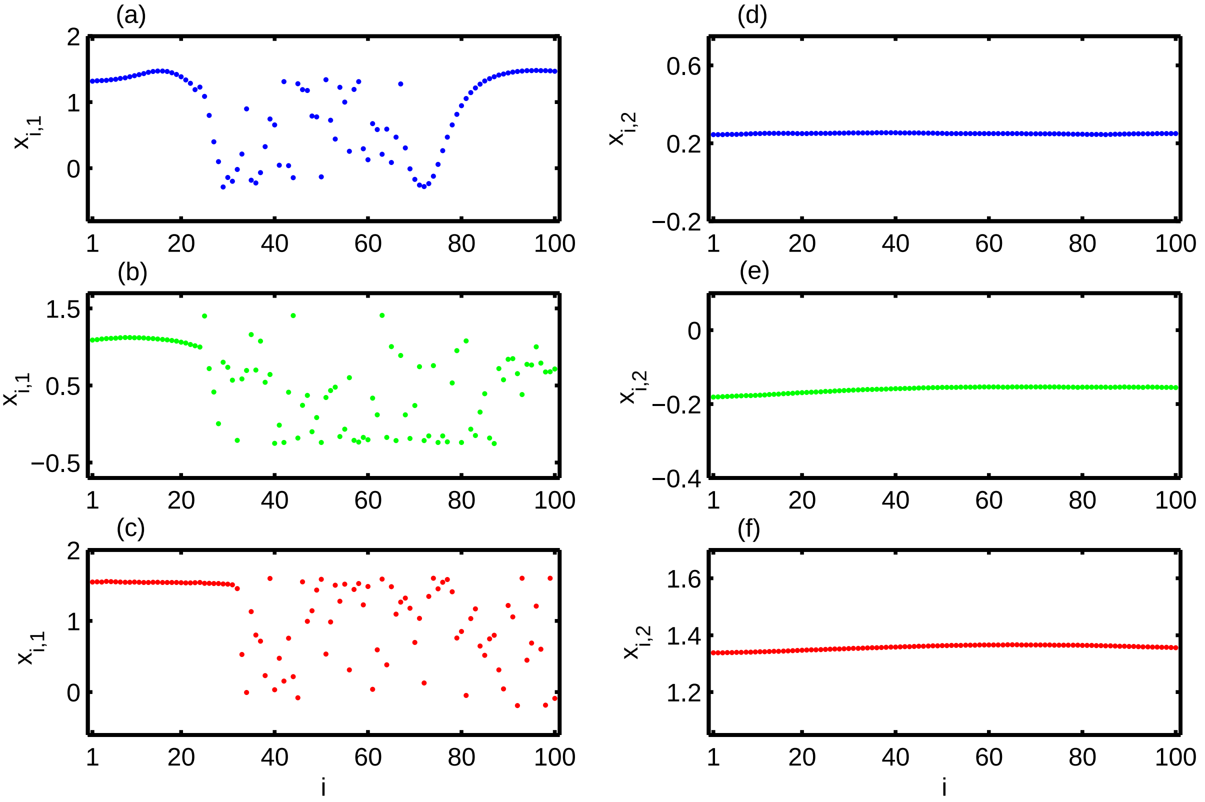

FIG. 1: Left panels show the snapshots of membrane potentials $x_{i,1}$ of the upper layer neurons ($i = 1, 2, ..., 100$) depicting chimera state for (a) $P = 10$ and $K_{ch} = 0.9$, (b) $P = 20$ and $K_{ch} = 1.1$, and (c) $P = 30$ and $K_{ch} = 1.1$. Right panels show the snapshots for the corresponding membrane potentials $x_{i,2}$ of the lower layer neurons in (d), (e) and (f) respectively. Here $K_{el} = 2.0$.

and lower layer neuron's membrane potentials with $K_{ch} = 0.9$ are shown in Figs. 1(a) and (d) respectively. Then we make $P = 20$ with the lower layer neurons in coherent state. For $K_{ch} = 1.1$, chimera pattern is seen in the upper layer of neurons. Snapshots of membrane potentials of both the layers are given in Figs. 1(b) and (e) respectively. Finally, taking $P = 30$, we detect chimera state in the upper layer while the lower layer is in coherent state. Snapshots for $K_{ch} = 1.1$ are shown respectively in Figs. 1(c) and (f).

II. **Chimera pattern in the proposed multi-layer structure in presence of inter layer heterogeneity**

In this section we are concerned with two layers having different dynamical natures of the

neurons by considering parameter mismatches. For this purpose, without loss of generality, we only change the parameter a that appears in the first two equations of both Eqns. (1) and (2). Then the equations governing the dynamics of the upper layer becomes

$$\begin{aligned} \dot{x}_{i,1} &= a_1 {x_{i,1}}^2 - {x_{i,1}}^3 - y_{i,1} - z_{i,1} + K_{ch}(v_s - x_{i,1})\Gamma(x_{i,2}(t)), \\ \dot{y}_{i,1} &= (a_1 + \alpha){x_{i,1}}^2 - y_{i,1}, \\ \dot{z}_{i,1} &= c(bx_{i,1} - z_{i,1} + e), \end{aligned} \tag{4}$$

and for the lower layer the equations are

$$\begin{aligned} \dot{x}_{i,2} &= a_2 {x_{i,2}}^2 - {x_{i,2}}^3 - y_{i,2} - z_{i,2} + K_{ch}(v_s - x_{i,2})\Gamma(x_{i,1}(t)) + K_{el} \sum_{j=1, j\neq i}^{N} E(x_{j,2}, x_{i,2}), \\ \dot{y}_{i,2} &= (a_2 + \alpha){x_{i,2}}^2 - y_{i,2}, \\ \dot{z}_{i,2} &= c(bx_{i,2} - z_{i,2} + e) \end{aligned} \tag{5}$$

All the parameters except a_1 and a_2 are taken same as above. As discussed in the article, for $a_1 = 2.8$, the individual neurons of the upper layer show square wave bursting (shown in Fig. 2(a)) and for $a_2 = 2.2$, the lower layer neurons exhibit plateau bursting (Fig. 2(b)), if no coupling is present.

Keeping $K_{el} = 1.5$ fixed and switching K_{ch} on, we observe that after being disordered, the upper layer experiences chimera pattern followed by synchronized state for increasing values of K_{ch}. Snapshot characterizing chimera pattern for $K_{el} = 1.5$ and $K_{ch} = 0.53$ can be seen in Fig. 2(c). Fig. 2(d) depicts the variation of strength of incoherence (SI) depending on K_{ch}. The regions I, II and III represent incoherent, chimera and coherent states respectively.

Next, we consider $a_1 = 2.8$ as before and $a_2 = 3.0$. For this choice, in absence of any interaction between the neurons, the neurons of the upper layer show square wave bursting (shown in Fig. 3(a)) and the lower layer neurons exhibit spiking, as shown in Fig. 3(b).

For fixed $K_{el} = 1.2$ and increasing K_{ch}, we observe that the upper layer experiences chimera pattern followed by synchronized state after being disordered initially. Snapshot depicting chimera state for $K_{el} = 1.2$ and $K_{ch} = 1.43$ is given in Fig. 3(c). Fig. 3(d) shows the variation of strength of incoherence (SI) depending on K_{ch}.

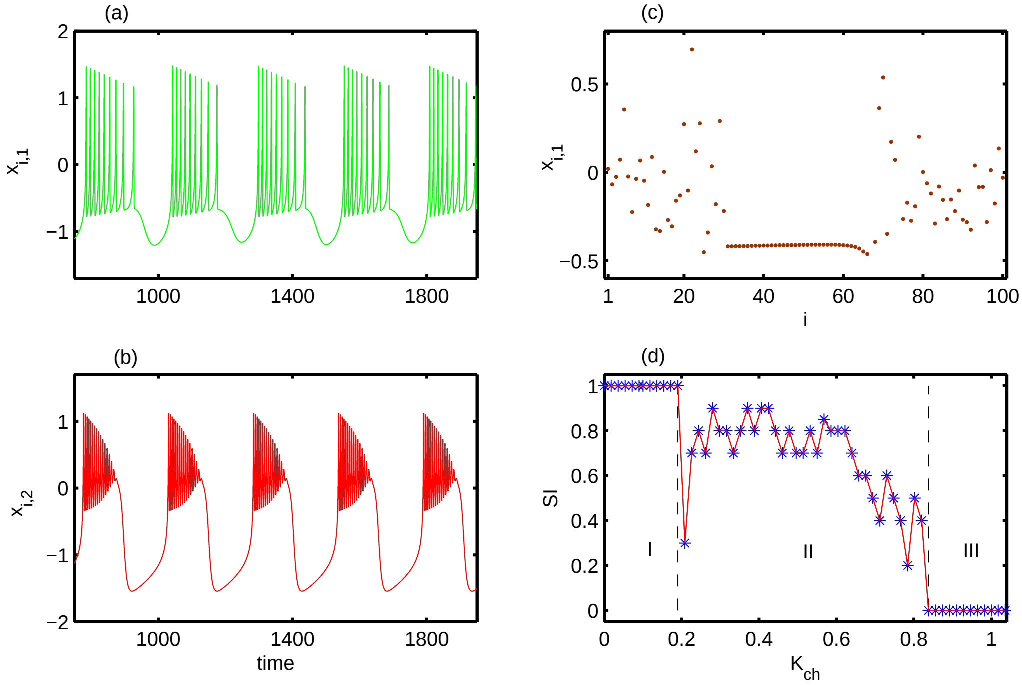

FIG. 2: Left panels show the time series of membrane potentials (a) $x_{i,1}$ exhibiting square wave bursting (for $a_1 = 2.8$) and (b) $x_{i,2}$ exhibiting plateau bursting (for $a_2 = 2.2$), $i = 1$. (c) Snapshot characterizing chimera state with $K_{el} = 1.5$ and $K_{ch} = 0.53$, (d) Variation of strength of incoherence (SI) by changing the inter-layer chemical synaptic coupling strength K_{ch} with $K_{el} = 1.5$.

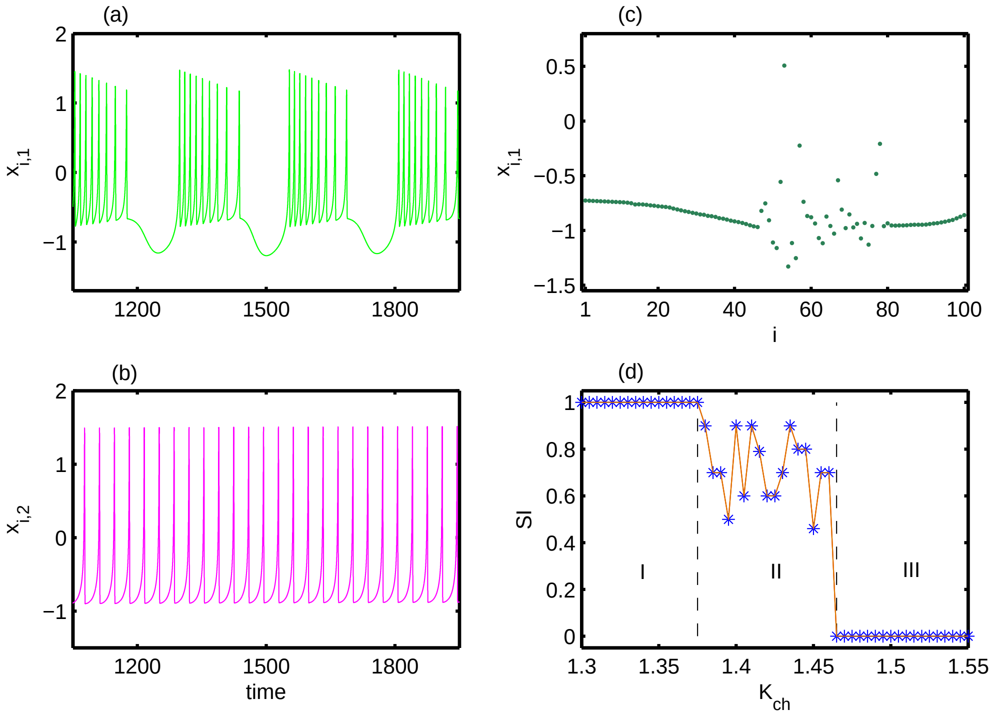

FIG. 3: Left panels show the time series of membrane potentials (a) $x_{i,1}$ exhibiting square wave bursting for $a_1 = 2.8$ and (b) $x_{i,2}$ exhibiting spiking for $a_2 = 3.0$ (here $i = 1$). (c) Snapshot characterizing chimera state with $K_{el} = 1.2$ and $K_{ch} = 1.43$, (d) strength of incoherence (SI) depending on K_{ch}. The regions I, II and III stand for incoherent, chimera and coherent states respectively.
